# Functional Endoscopic Sinus Surgery in Management of Pott's Puffy Tumor in a Pregnant Woman: A Clinical Case Report and Literature Review

**DOI:** 10.1002/ccr3.70087

**Published:** 2025-01-07

**Authors:** Seyed Ali Dehghan Manshadi, Azin Tabari, Hana Saffar, Faeze Salahshour, Nahid Shafiee, Azam Sadat Izadpanah, Seyed Mohammad Piri

**Affiliations:** ^1^ Department of Infectious Disease and Tropical Medicine, Iranian Research Center for HIV/AIDS (IRCHA) Tehran University of Medical Sciences Tehran Iran; ^2^ Otorhinolaryngology Research Center, IKHC Tehran University of Medical Sciences Tehran Iran; ^3^ Cancer Research Institute, IKHC Tehran University of Medical Sciences Tehran Iran; ^4^ Department of Radiology, Advanced Diagnostic and Interventional Radiology Research Center (ADIR) Tehran University of Medical Sciences Tehran Iran; ^5^ Liver Transplantation Research Center, Imam‐Khomeini Hospital Tehran University of Medical Sciences (TUMS) Tehran Iran; ^6^ Department of Infectious Disease and Tropical Medicine Tehran University of Medical Sciences Tehran Iran

**Keywords:** adults, endoscopic surgeries, intracranial, osteomyelitis, Pott's puffy tumor, pregnancy

## Abstract

PPT is a life‐threatening intracranial complication, which is essential to be considered in both children and adults presenting with sinusitis symptoms.

## 1 Introduction

Skull bone osteomyelitis is a rare condition frequently affecting children aged 6–15. Frontal bone osteomyelitis as a complication of chronic untreated sinusitis, forehead trauma, or after sinus surgeries and dental procedures is responsible for about 2% of all children's osteomyelitis [1, 2]. Pott's puffy tumor (PPT) is an uncommon phenomenon characterized by frontal bone osteomyelitis with associated sub‐periosteal abscess and swelling following an untreated frontal sinusitis, which leads to fever, severe headache, periorbital edema, and nasal contestation [3].

You should immediately evaluate patients suspected of PPT using computed tomography (CT) or magnetic resonance imaging (MRI). These imaging can determine the existence of any intracranial complication that can compromise the outcome. Broad‐spectrum intravenous antibiotic therapy should start as soon as the diagnosis has been confirmed. Based on the microbiological study, gram‐positive organisms are the most responsible agents in PTT; therefore, penicillin, third‐generation cephalosporin, vancomycin, and metronidazole for 4–8 weeks are the choices [4, 5].

Along with antibiotic therapy, surgical interventions are necessary to drain the affected sinus and resect the damaged bone. Two types of surgery have been conducted in this matter. Traditionally, open surgery was performed to evaluate the sinus under direct vision, but it ended with a bad cosmetic outcome. Recently, endoscopic surgeries such as functional endoscopic sinus surgery (FESS) have been introduced, with lower morbidity and mortality rates and a better cosmetic outcome [4, 6, 7].

In this study, we have presented a pregnant adult with PPT without any prior history of trauma or sinus manipulation, which has been managed with FESS procedure and had a favorable outcome.

## 2 Case History/Examination

**FIGURE 1:**
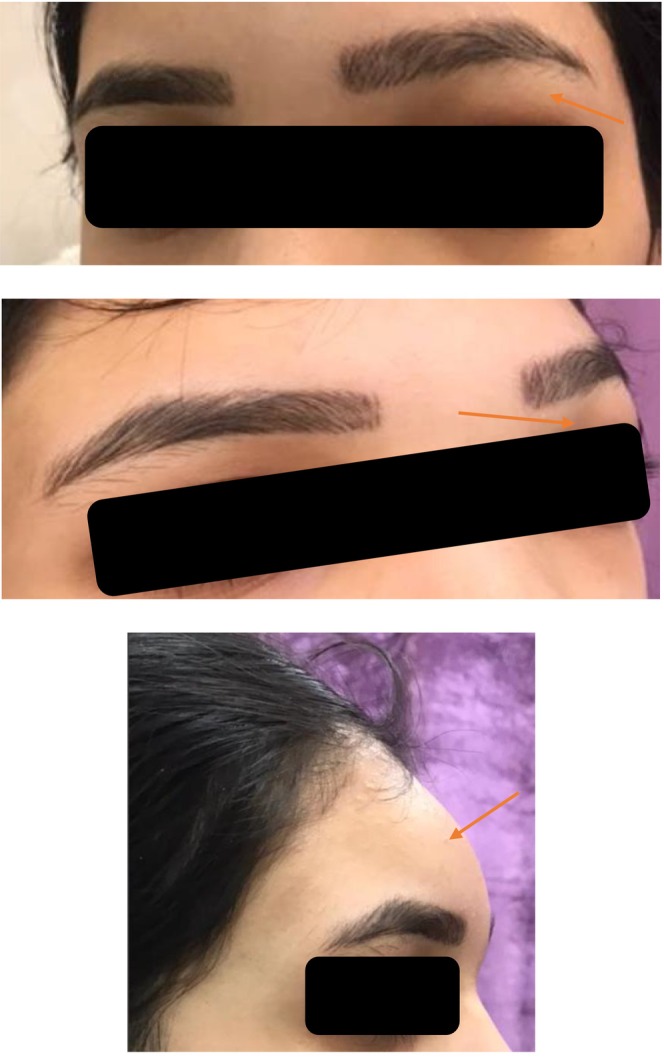
Frontal swelling and periorbital edema.

A 27‐year‐old woman at 25 weeks' gestation presented with a history of frontal headaches and swelling, periorbital edema, and pain. She was admitted to the infectious diseases ward (Figure 1). She had suffered from these symptoms for the past 1 month following a common cold, which had been treated in ambulatory clinics. She reported mild anemia from prenatal evaluations, and no other diseases were noted. The patient denied any kind of head trauma, dental procedures, previous sinus surgery, and insect bites.

## 3 Methods

On admission, primary vital signs were all within normal ranges. Her first laboratory analysis results were indicative of a normal white blood cell count (WBC) of 7.9 × 109/L, low hemoglobin (6.4 g/dL), increased erythrocyte sedimentation rate (ESR), and C‐reactive protein (CRP) of 115 mm/h and 32 mg/dL, respectively.

**FIGURE 2:**
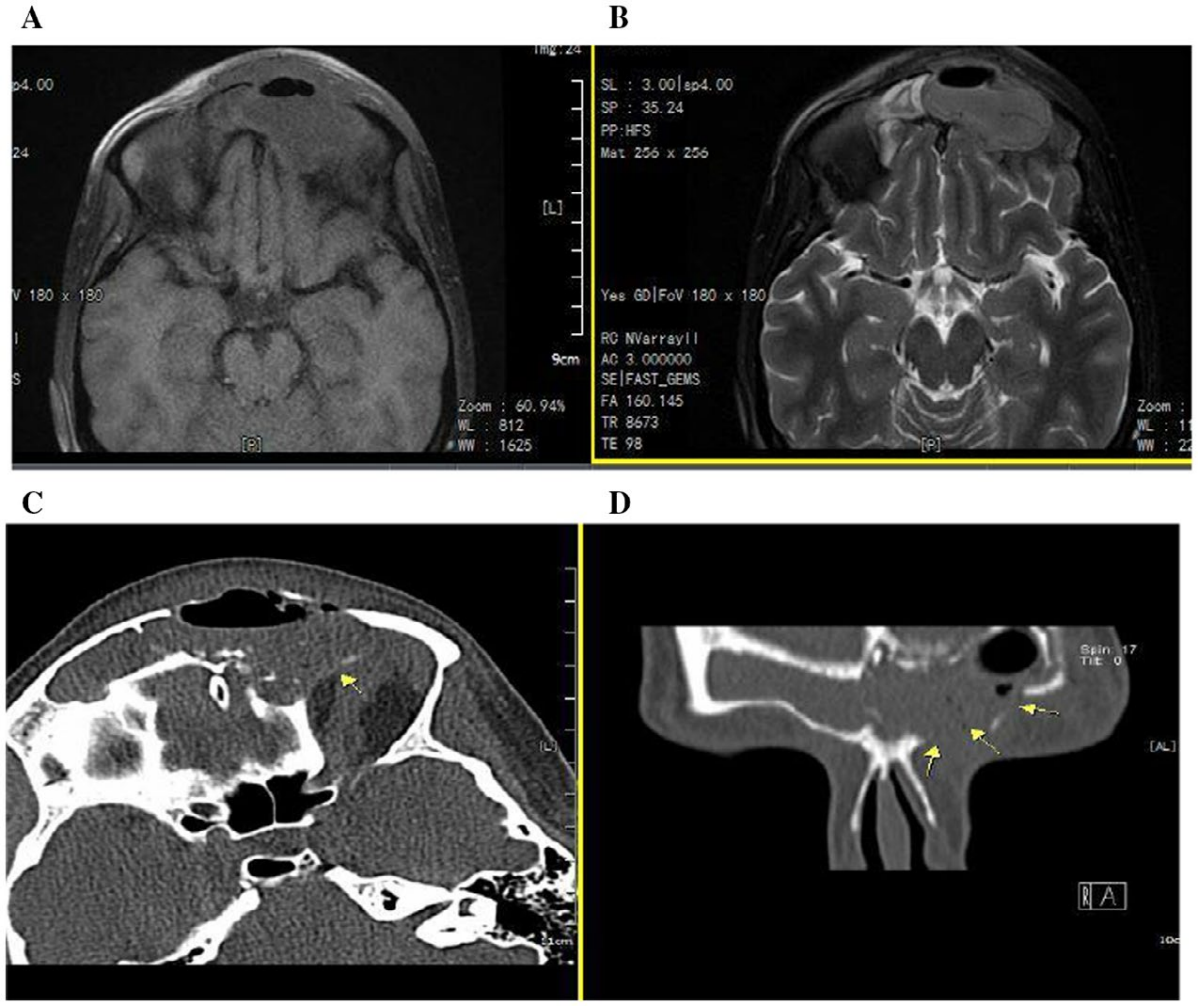
Brain MRI. (A and B) In MRI images, para nasal sinuses were depicted expansion and opacification of the left frontal sinus. (C and D) The anterior and posterior sinus wall have bowing toward the left orbit and frontal scalp associated with disruption (yellow arrows) of bony sinus walls and inflammatory changes in the left frontal scalp.

The patient was started on intravenous antibiotics, including ceftriaxone and clindamycin, based on the clinician's judgment and empirical treatment approach. The MRI revealed a hypointense signal in the frontal sinus and hypointense soft tissue swelling overlying the frontal bone, consistent with inflammation and potential abscess formation. Pott's puffy tumor was confirmed as a complication of frontal sinusitis without brain involvement (Figure 2). The patient's antibiotics were changed to vancomycin (15–20 mg/kg every 8–12 h) and meropenem (1–2 g every 8 h) following the confirmed diagnosis, based on the results of the antibiogram susceptibility test. After careful obstetrical evaluation and normal ultrasonography of the fetus, based on ophthalmologic and ENT consultations, the patient was a candidate for functional endoscopic sinus surgery (FESS).

## 4 Conclusion and Results

**FIGURE 3:**
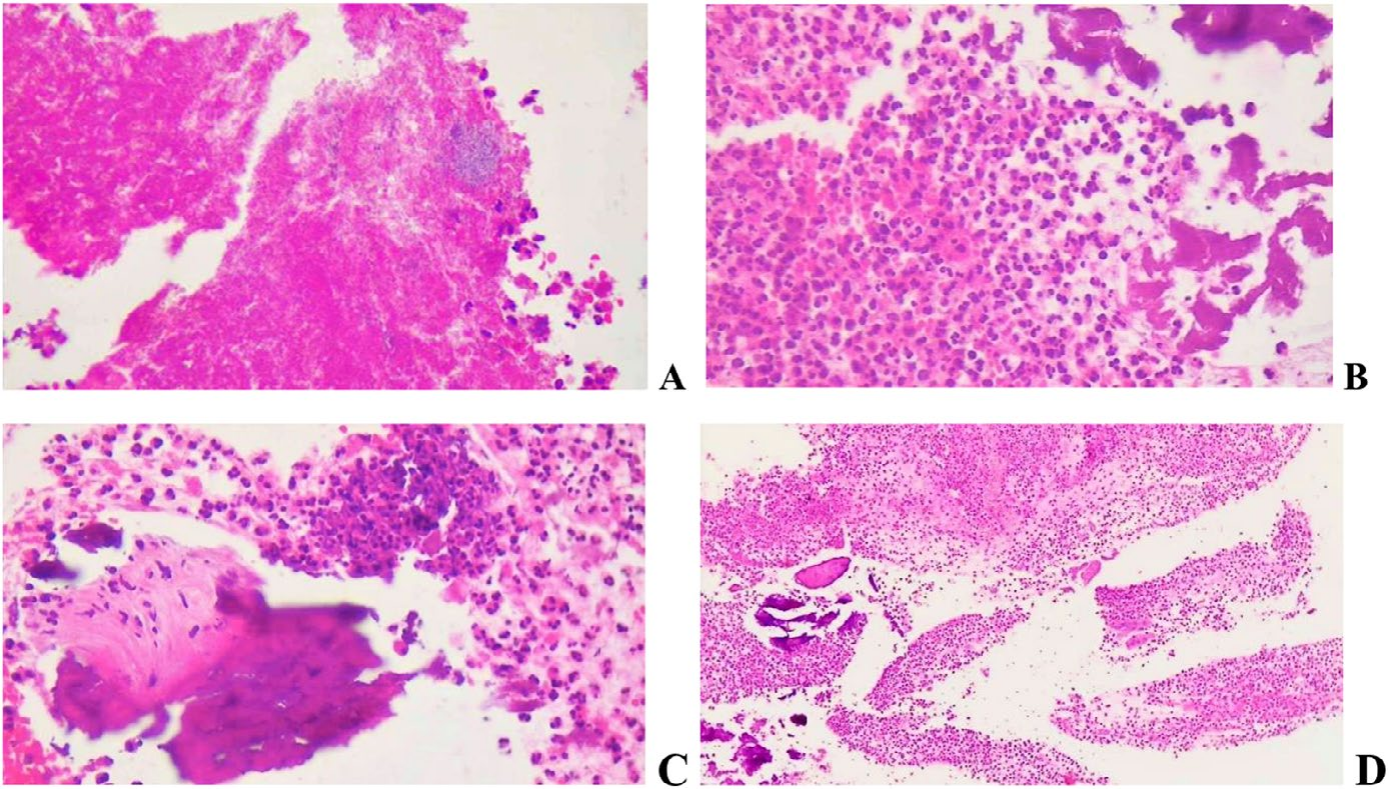
Hematoxylin and eosin stain (H&E) of a pathological specimen. (A) ×40 magnification, Hematoxylin and eosin stain. Sections show dense infiltration of inflammatory cells, predominantly composed of neutrophils, associated with necrosis and bacterial colonization. (B and C) Fragments of dead necrotic bone trabeculae are also present. (D) ×400 magnification, multinucleated giant cells in a background of severe acute inflammation, areas of necrosis and bacterial colonization, and necrotic bone associated with acute inflammation.

The patient underwent the FESS surgery successfully, and the sinus specimen was sent for microbiology and pathology studies. *Staphylococcus epidermidis* was detected on microbiological examination of the specimen and the pathological report was in favor of congestion and necro‐inflammation of the right frontal lesion (Figure 3).

Due to the rarity of our patient's condition, complete immune–based laboratory analyses were performed. Complement deficiencies and other immune dysfunction situations were ruled out.

After 6 weeks of intravenous antibiotic therapy (vancomycin and meropenem), the patient was discharged in healthy condition with normal laboratory results and normal fetal ultrasonography findings. On her follow‐up sessions, she did not complain of any symptoms at 36 weeks' gestation, and a normal delivery was conducted later at her due time.

PPT is a rare and life‐threatening intracranial complication in the frontal sinus, which is essential to be considered in both children and adults presenting with fever, headache, forehead swelling, periorbital edema, and nasal congestion with a history of previous sinusitis, forehead trauma, or other head and neck surgical procedures.

## 5 Discussion

PPT is a rare life‐threatening complication of frontal osteomyelitis due to previous chronic sinusitis, forehead direct or indirect trauma, sinus surgeries, dental procedures, and rarely, insect bites. After proper imaging in patients with associated symptoms, immediate medical and surgical treatment is essential to prevent severe intracranial complications and death [8].

Despite the low incidence of frontal osteomyelitis in the overall population, children and adolescents are more likely to be diagnosed with PPT, with an incidence rate of 72% of all intracranial complications. However, recently, due to broad antibiotic resistance and vast imaging evaluations, adults have been considered to account for PPT increasingly [9, 10] as we have seen in our patient with mild sinusitis, which has progressed to PPT in less than a month despite outpatient antibiotic therapy.

PPT has been reported previously in pregnant women, similar to our patient. Based on the literature, it is essential to manage intracranial complications in pregnant patients with multidisciplinary consults to avoid any complications for both the mother and her child [11]. Therefore, we evaluated our patient with different consults from gynecology, infectious diseases, ophthalmology, and ENT departments.

PPT etiology has been mostly considered a polymicrobial infection. The most common organisms are believed to be non‐enterococcal streptococci. However, in our patient, *S. epidermidis* was responsible for the infection, which has rarely been mentioned in previous studies [4, 12].

Similar to the literature, MRI was used in our patient for diagnosis confirmation and evaluation of intracranial involvement. The treatment consisted of a combination of broad‐spectrum antibiotics and endoscopic surgery to drain the sinus and resect the involved bone. Our patient was given vancomycin and meropenem for 6 weeks due to the vast antibiotic resistance to other treatment choices in our country [2, 13]. Fortunately, immediate and proper treatment as well as the absence of brain damage have caused the best outcome expected in our patient.

## Author Contributions

**Seyed Ali Dehghan Manshadi:** supervision, validation. **Azin Tabari:** supervision, validation. **Hana Saffar:** validation. **Faeze Salahshour:** validation. **Nahid Shafiee:** writing – original draft. **Azam Sadat Izadpanah:** writing – original draft. **Seyed Mohammad Piri:** resources.

## Consent

Written informed consent was obtained from the patient to publish this report in accordance with the journal's patient consent policy.

## Conflicts of Interest

The authors declare no conflicts of interest.

## Data Availability

The authors confirm that the data supporting the findings of this study are available within the article.
